# Effect of the Addition of Al-5Ti-0.25C and Annealing on the Mechanical Properties of Open-Cell Al Foams

**DOI:** 10.3390/ma18092147

**Published:** 2025-05-07

**Authors:** Omar Novelo-Peralta, Manuel Farid Azamar, Julio Esteban Méndez Durán, Yessica Lizbeth Ávila, Antonio Enrique Salas Reyes, Ramiro Bazáez, Ignacio Alejandro Figueroa, Gabriel Ángel Lara Rodríguez

**Affiliations:** 1 Instituto de Investigaciones en Materiales, Universidad Nacional Autónoma de México, Ciudad Universitaria, Del. Coyoacán, Ciudad de México 04510, Mexico; omarnovelo@iim.unam.mx (O.N.-P.); manuel.azamar.j@comunidad.unam.mx (M.F.A.); jemendez@pceim.unam.mx (J.E.M.D.); yavila@pceim.unam.mx (Y.L.Á.); 2 Departamento de Ingeniería Metalúrgica, Facultad de Química, Univesidad Nacional Autónoma de México, Ciudad de México 04510, Mexico; enriquesalas@comunidad.unam.mx; 3 Departamento de Obras Civiles, Universidad Técnica Federico Santa María, Valparaíso 2390123, Chile; ramiro.bazaez@usm.cl

**Keywords:** open-cell metallic foam, aluminum alloy, grain refinement, annealing, energy absorption capacity

## Abstract

Commercially pure aluminum (Al) was refined through the addition of the Al-5Ti-0.25C master alloy, resulting in the formation of Al_3_Ti and TiC phases, which serve as refining agents. Open-cell metallic foams were successfully produced using the replication casting technique, with pore sizes ranging from 1.00 to 3.35 mm. For the infiltration process, refined aluminum was used, while unrefined aluminum served as a baseline reference. The resultant foams underwent multiple annealing cycles at 480 °C, with the most refined and homogeneous microstructure observed after 504 h. Comprehensive microstructural characterization was conducted utilizing scanning electron microscopy and optical microscopy. Additionally, uniaxial compression tests were performed to generate stress–strain profiles for the foams, facilitating an assessment of their energy absorption capacity. The findings indicated an enhancement in energy absorption capacity by a factor of 2.4 to 3, which can be attributed to the incorporation of Al-5Ti-0.25C and the subsequent annealing process.

## 1. Introduction

Open-cell metallic foams constitute a family of porous metals formed by a continuous metallic matrix coexisting with an interconnected porous volume. Their functionality has been extensively studied within a wide range of possible applications [1,2,3], as they combine the properties of metals, e.g., mechanical strength and thermal/electrical conductivity, with the advantages of a lightweight-porous structure. Aluminum foams serve a variety of multifunctional applications across several industries. Key uses of aluminum foams include non-flammable construction materials for acoustic and thermal insulation, mechanical damping, sound absorption, sandwich cores, and lightweight panels designed for the construction and transportation sectors. These materials are engineered to withstand buckling and impact while also providing vibration control and deformation isolation [4,5]. Nowadays, investigation related to open-cell metallic foams is focused on the development of novel methods to increase their mechanical properties, such as the addition of reinforcing particles [6,7] or alloying elements [8], the application of reinforcing coatings [9,10], as well as through heat treatments [11,12]. Except for reinforcing coatings, the mechanisms mentioned above involve inducing the presence of discontinuities or defects within the crystalline lattice, which hinders the movement of dislocations.

Previous research concerning the strengthening mechanisms for metals has demonstrated that the effect on the final mechanical properties attained after processing depends on the nature and amount of the formed crystalline discontinuities/defects. Similar results have been found for open-cell metallic foams. Azamar et al. [8] reported an important increment of strength and toughness resulting from the Cu addition to open-cell Al foams without subsequent heat treatments. Such increment was attributed to θ-Al_2_Cu dendrites throughout the α-Al matrix. Zhou et al. [11] studied the heat treatment of open-cell 6101 aluminum foams. Their findings indicate that both annealing and T6 strengthening techniques effectively optimize the mechanical and deformation properties of these foams. These enhancements were highly dependent on the resulting microstructure, making them suitable for various engineering applications.

Similarly, Casati and Vedani [6] described the most extensively used strengthening mechanisms for metal matrix composites (MMCs), essentially consisting of the addition of reinforcing particles, which can also act as grain refining agents. Nevertheless, these mechanisms are often limited to solid-state processes due to the low wettability of the usually employed non-metallic particles (typically ceramic-based) with the molten metal matrix. Based on this, current literature on grain refinement as a mechanism for enhancing the strength of metallic foams remains limited.

Grain refinement is commonly used to improve the strength and plasticity of metallic materials, which is obtained by changing the size of grain structure [13], followed by a microstructural homogenization obtained from a recrystallization process [14]. Several techniques have been developed to achieve grain refinement, i.e., grain refining by vibration and stirring during solidification [15], rapid solidification, adding a grain refiner agent, and severe plastic deformation [13]. Considering the metallic nature of open-cell metallic foams, grain refinement is a plausible way to increase their mechanical properties. Wang et al. [16] refined Mg foams produced through the replication method by multi-axial forging, reporting an important increment in yield strength and plastic collapse strength without pore structure modification. Lehmhus et al. [17] refined AlSi7 foams produced through powder metallurgy by varying solidification rates and adding Sr, B, and TiB_2_/TiAl_3_ as refiner agents, finding that whilst grain refinement based on TiB_2_/TiAl_3_ additions may prove a valuable alternative, the addition of Sr and B revealed detrimental effects on pore structure.

Open-cell Al foams are of singular relevance within the family of metallic foams due to their overall properties and easy handling of Al. Considering that Al-based alloys are usually refined by adding Al-Ti compounds [18], this investigation evaluates the impact of grain refinement on the microstructure of open-cell aluminum foams by adding an Al-5Ti-0.25C refining agent. Additionally, it examines the influence of annealing on the energy absorption capacity of these foams. The hypothesis proposes that Al-5Ti-0.25C particles serve as effective pinning and nucleation sites, inhibiting or stopping grain growth and enhancing the material strength [6].

## 2. Materials and Methods

### 2.1. Fabrication of Refining Agent Al-5Ti-0.25C

The Al-5Ti-0.25C master alloy was produced using Al with a purity of 99.9% and Ti with a purity of 99.5%. A total of 142.125 g of Al and 7.5 g of Ti were weighed for the alloying process. The melting process was carried out using an Edmund Bühler MAM-1 electric arc furnace (Bodelshausen, Germany), and the resulting alloy was cast into a hollow rod-like shape. Following this, 0.375 g of carbon flakes, averaging 300 nm in size, were uniformly distributed along the length of the hollow rod. The carbon was then diffused at 1000 °C by placing the Al-Ti rod into a Lindberg tubular furnace (Riverside, MN, USA) with a constant Ar flow.

### 2.2. Fabrication of Refined Open-Cell Al Foams

Refined Al cylindrical ingots were produced using an EMA 40 KVA induction furnace (Petersbergstr, Germany), melting 1.973 g of Al-5Ti-0.25C master alloy per 500 g of Al alloy 1100 under Ar atmosphere conditions. The studied open-cell foams were produced by the replication casting technique [19,20,21], using NaCl particles as a preform and refined Al as the load for infiltration. In addition, un-refined Al open-cell foams were produced as a reference. The NaCl particles were sieved using meshes to obtain two pore sizes: (P1) 1.00 to 2.00 mm and (P2) 2.38 to 3.35 mm. The replication casting process was carried out in a Carbolite resistance furnace (Hope, UK) at 780 °C. For the process, the Ar pressure was held at 0.5 kg cm^−2^ for 1 h. Subsequently, such pressure increased to 1.5 and 1.25 kg cm^−2^ for pores P1 and P2, respectively, for 25 min to permeate the NaCl preform with the molten metal. After the infiltration, the crucible was extracted from the furnace and placed over a Cu block to induce the solidification of the molten metal from bottom to top. The composite Al (Al-5Ti-0.25C)-NaCl was extracted from the crucible and machined into cylindrical samples of 2.00 cm in length and 2.54 cm in diameter. Finally, the NaCl preform was leached in distilled water using an ultrasonic bath, producing highly porous Al (Al-5Ti-0.25C) foams. The recrystallization temperature was determined utilizing scanning differential calorimetry. In order to determine the optimal heat treatment parameters for achieving a refined and homogeneous microstructure, annealing was conducted in a Carbolite furnace over varying heat treatment times, i.e., 168, 336, 504, 672, and 840 h.

### 2.3. Microstructural and Mechanical Characterization

Grain refinement in open-cell aluminum foams was studied using advanced microscopy techniques. Scanning electron microscopy was performed with a Benchtop SEM JEOL JCM-6000 (Tokyo, Japan), while optical microscopy analysis was carried out using an Olympus Vanox AHMT3 microscope (Tokyo, Japan). The foam samples were prepared according to standard metallographic procedures to facilitate the microstructural analysis. A quantitative assessment of the grain structure was carried out through image processing using the commercial software ImageJ (Version 1.54j).

Uniaxial compression tests were performed to generate stress–strain plots for the refined and unrefined foams. These tests were carried out using the universal mechanical testing machine, Instron 1125-5500R (Norwood, MA, USA), at a strain rate of 4.1 × 10^−4^ s^−1^. The resulting graphs enabled the calculation of energy absorption capacity. For statistical purposes, each testing condition involved six samples with dimensions of 25 mm in diameter and 20 mm in length.

## 3. Results and Discussion

**Figure 1:**
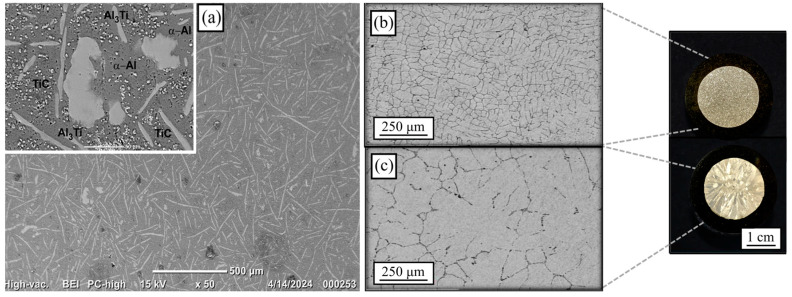
Microstructure of (**a**) Al-5Ti-0.25C master alloy, (**b**) refined Al, and (**c**) unrefined Al.

The microstructure of the obtained Al-5Ti-0.25C master alloy is shown in Figure 1a. A uniform distribution of the acicular Al_3_Ti intermetallic throughout the Al matrix was observed.

The subsequent analysis identified the presence of TiC, which, alongside Al_3_Ti, functions as an effective grain refining agent. This mechanism is attributable to the significantly higher melting points of TiC and Al_3_Ti, approximately 1360 °C [22], and 660 °C, respectively, in comparison to aluminum (Al). These higher melting points facilitate their role as nucleation sites during the solidification process of the alloy. Wang et al. [18] reported similar results for Al-5Ti-0.25C produced using a melt reaction method in a high-frequency induction furnace, unlike the carburization of the Al-Ti rod described in this work. Yücel Birol [23] identified that an excess of Ti contributes significantly to the grain structure refinement when utilizing titanium carbide (TiC) grain refiners, as it effectively hinders grain growth. The microstructure of the refined Al after adding Al-5Ti-0.25C is shown in Figure 1b. In addition, the microstructure of unrefined Al is also presented in Figure 1c as a reference. In both cases, the microstructure is mainly constituted by a dendritic equiaxed arrangement. Nevertheless, from visual examination, a significant microstructural refinement is appreciated with the Al-5Ti-0.25C addition, bearing in mind that such microstructure was achieved in the as-cast condition, i.e., prior recrystallization process. Refined Al was considered a plausible alternative to produce strengthened open-cell Al foams based on these outcomes.

**Figure 2:**
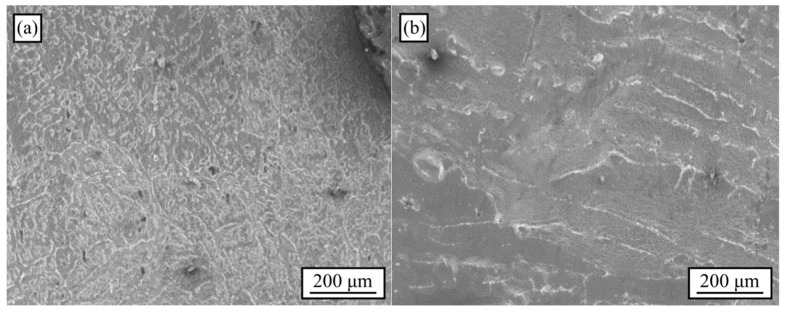
Microstructure of the open-cell foams produced using (**a**) refined Al and (**b**) unrefined Al.

Figure 2 shows the microstructure of open-cell foams produced using refined (Figure 2a) and unrefined Al (Figure 2b).

These micrographs were taken as-cast, i.e., after solidification and the subsequent samples metallographic preparation. Similar to bulk metal, refined and unrefined open-cell Al foams presented a dendritic microstructure, with the grain refinement prevailing, derived from the addition of Al-5Ti-0.25C. The effect of NaCl particles on the as-cast microstructure of open-cell Al foams was considered negligible [8] due to the low thermal conductivity of NaCl [24]. Therefore, the overall microstructural variation was attributed to the addition of the grain refiner agent for both pores P1 and P2, despite their pore size differences.

**Figure 3:**
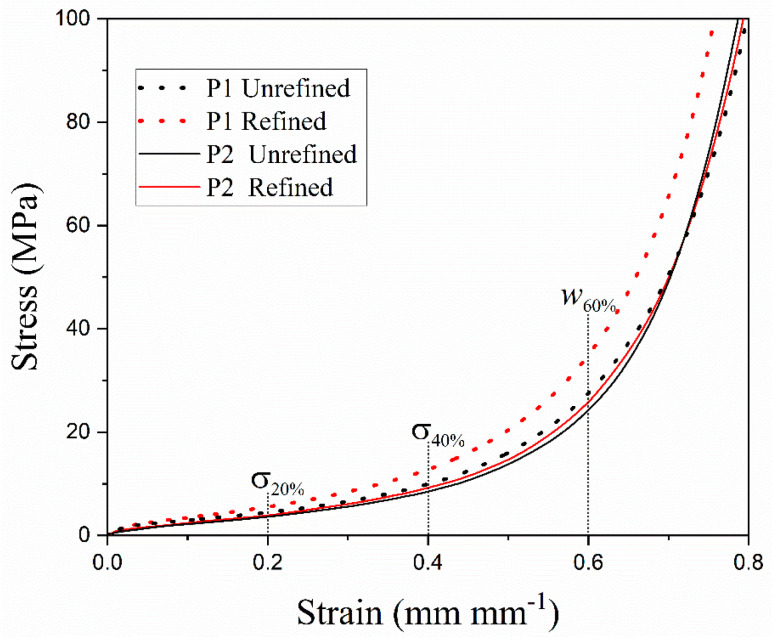
Stress–strain plots of the as-cast refined and unrefined open-cell Al foams.

Figure 3 shows the stress–strain plots corresponding to the as-cast refined and unrefined open-cell Al foams of pore P1 and P2.

All plots presented the typical compressive behavior for this material, i.e., firstly, a linear elastic zone, then an extended plateau, and finally, the densification of the foam [25]. Figure 3 also shows that the refined samples displayed an increment in strength throughout the entire strain axis for both pores. The observed increment can be primarily attributed to the enhanced microstructural characteristics resulting from the incorporation of Al-5Ti-0.25C. This improvement is driven by the presence of solid TiC and Al_3_Ti particles during the solidification process of molten Al. The development of this structure results from the cooling of the reactor/crucible to room temperature, which facilitates the formation of the foams, as detailed in the work by Osorio et al. [19]. The addition of the refiner, along with the cooling rate associated with the technique and infiltration process, significantly influenced the development of refined structures in metallic foams. These particles are crucial in determining the final grain size of metal matrix materials, as they interact with grain boundaries, serving as pinning points that impede or halt grain growth [4].

Moreover, the TiC and Al_3_Ti particles reinforced the Al matrix in a mechanism similar to that described by Casati and Vedani [6]. The Al_3_Ti phase enhances grain refinement, while the presence of TiC particles inhibits grain growth in the refined foam, remaining even after the manufacturing process. These particles serve as barriers to dislocation movement, thereby contributing to an improvement in mechanical properties. Additionally, the process of grain refinement results in an increased density of grain boundaries, which also act as obstacles to dislocation movement. This phenomenon not only enhances mechanical strength but also reduces ductility. The second region of the stress–strain curve for metal foams is associated with the plastic deformation of the pore walls. The hardening effect causes a decrease in the wall ductility, which is manifested in the shortening of the plateau. For this reason, the plateau was slightly shorter in the refined open-cell Al foams since an increase in strength is usually followed by a decrease in ductility, which generates an accelerated collapse of the cell walls and, consequently, reduces the plateau region in the stress–strain curves.

**Figure 4:**
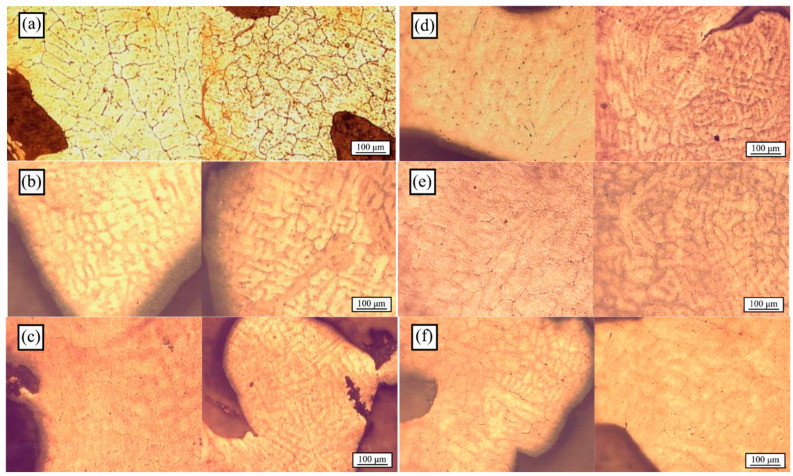
Microstructure of the unrefined (right) and refined (left) open-cell Al foams by the addition of Al-5Ti-0.25C after annealing at 480 °C for (**a**) 0 h, (**b**) 168 h, (**c**) 336 h, (**d**) 504 h, (**e**) 672 h, and (**f**) 840 h.

The microstructures of the annealed foams at 480 °C obtained by optical microscopy are shown in Figure 4, for 0 h (Figure 4a), 168 h (Figure 4b), 336 h (Figure 4c), 504 h (Figure 4d), 672 h (Figure 4e), and 840 h (Figure 4f).

**Figure 5:**
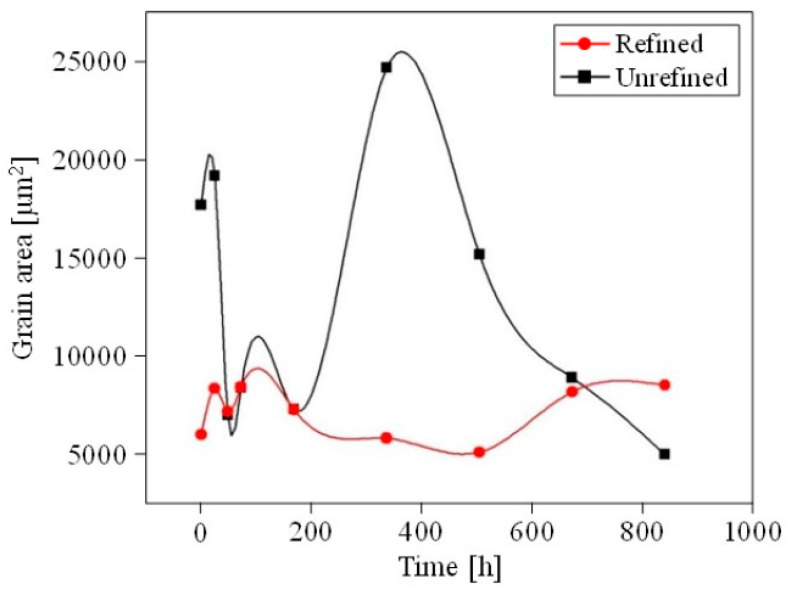
Estimated grain size as a function of annealing time at 480 °C.

The grain size did not present significant variation after annealing for 168 h between refined and unrefined foams, which means that this time was insufficient to achieve the Al recrystallization. On the other hand, after annealing for 336, 504, and 672 h, the foam grain refinement with Al-5Ti-0.25C was evident, even prior to quantitative analysis. After annealing for 840 h, grains of refined foams were enlarged, and the average grain size increased, which was attributed to an excessive heat treatment time, thereby causing undesired grain growth. However, the observed grain size after the annealing of refined foams for all heat treatment times was significantly smaller (Figure 2a) when compared to those of the unrefined as-cast foams (Figure 2b). Figure 5 shows the grain size as a function of annealing time at 480 °C for refined and unrefined open-cell Al foams.

**Figure 6:**
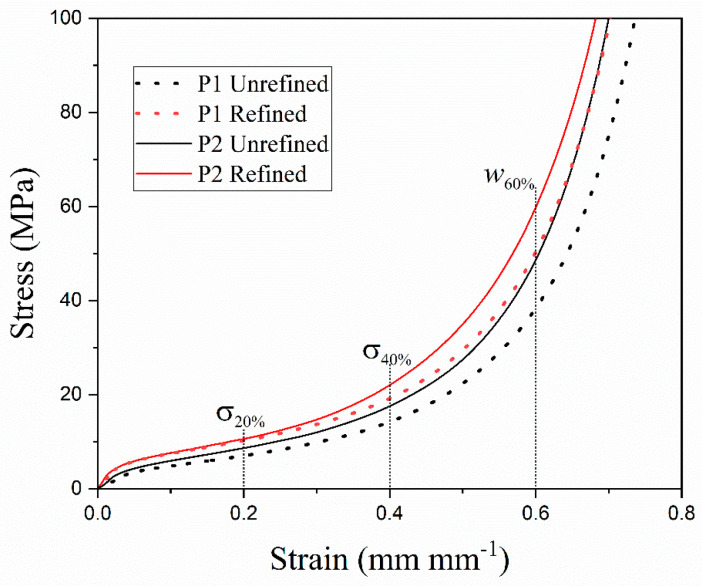
Stress–strain plots of the refined and unrefined open-cell Al foams after annealing at 480 °C for 504 h.

The average grain size was estimated from 100 measurements of the studied surface area. Before annealing, the refined samples presented a much smaller average grain size than the unrefined ones. After 168 h of annealing, the estimated grain size for refined foams decreased, reaching its minimum at 504 h, whilst the grain size for unrefined foams increased, reaching its maximum at 336 h. In contrast, after 840 h of annealing, the average grain size peaked for the refined foams, while it exhibited its lowest value for unrefined foams. Notably, the optimal microstructure was achieved following an annealing period of 504 h, which is considered ideal for the annealing of refined open-cell aluminum foams. The samples subjected to these specific conditions were subsequently evaluated through compression tests, as it is commonly understood that a finer grain size correlates with enhanced mechanical properties [26]. Figure 6 shows the stress–strain plots corresponding to the refined and unrefined open-cell Al foams after annealing at 480 °C for 504 h, corresponding to pore P1 and P2 samples.

From this figure, the strength of the foam notably increased due to Al recrystallization. The importance of annealing lies in the formation of a fine and homogenized microstructure (Figure 4d). From this figure, an increment in toughness was also observed, indicating the strengthening of the cell walls.

**Figure 7:**
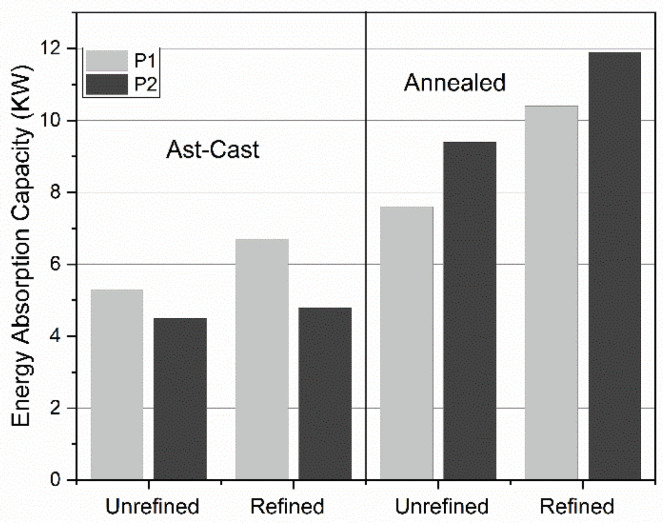
Energy absorption capacity *W* (MJ m^−3^) for the refined and unrefined as-cast and annealed open-cell Al foams at 480 °C and 504 h.

Figure 7 illustrates the energy absorption capacity *W* (in MJ m^−3^) of both refined and unrefined as-cast and annealed open-cell aluminum foams. This absorption capacity was calculated as the area under the curve between 0% and 60% strain, in accordance with the defined methodology for assessing energy absorption capacity, i.e., [19]:

$$W=\int_{0}^{\varepsilon }\sigma d\varepsilon$$

**Figure 8:**
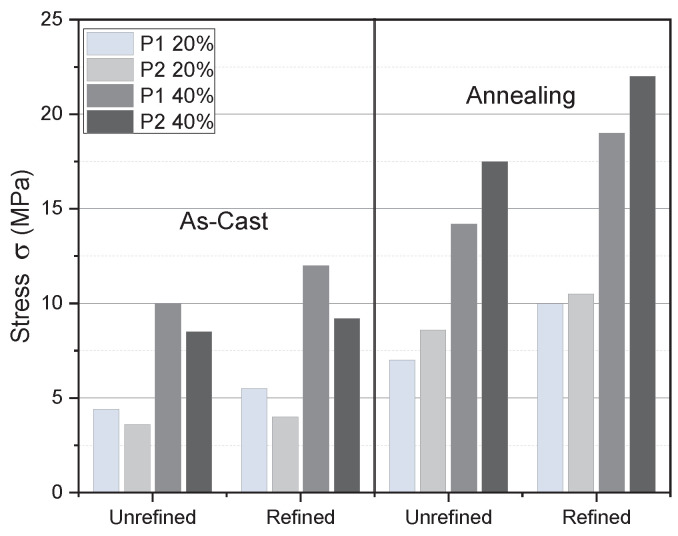
Stress *σ* (in MPa) at 20% and 40% of strain for the refined and unrefined as-cast and annealed open-cell Al foams at 480 °C and 504 h.

The relationship between the average energy absorption capacity of the refined-annealed and the as-cast-unrefined foams is 30% and 16% for pores P1 and P2, respectively. Such increment is significant, considering that the energy absorption capacity governs open-cell metallic foams’ damping and crash dissipation response. As the increment in toughness and strength prevailed despite the pore size, such increment was attributed to the grain refinement effect and the Al recrystallization. It is worth mentioning that the foams are fully functional before the collapse of the cell structure, i.e., before the densification, which occurs between 20% and 40% of strain. Figure 8 shows the stress *σ* (in MPa) at 20% and 40% of strain for the refined and unrefined as-cast and annealed open-cell Al foams.

Figure 8 shows a notable average increase of 17% in mechanical strength attributable exclusively to the addition of the Al-5Ti-0.25C refining agent in the as-cast foams. In contrast, the annealed foams exhibited a more substantial average increase of 28% in mechanical properties, regardless of whether they were refined or unrefined. It is important to highlight that the enhancement in properties achieved through grain refinement via the addition of Al-5Ti-0.25C, combined with subsequent annealing, was particularly significant. The strain ratios observed in refined-annealed foams compared to cast-unrefined foams at 20% strain are 2.75 for pore P1 and 3.66 for pore P2. At 40% strain, these ratios adjust to 2.0 for pore P1 and 3.75 for pore P2. These findings can be attributed to the higher density of grain boundaries in the refined-annealed foams, which serve as obstacles to dislocation movement. This phenomenon results from the varying orientations of adjacent grains coupled with the significant lattice disorder characteristic of these regions, hindering the ability of the dislocations to move along a continuous slip plane [6].

## 4. Conclusions

- Aluminum (Al) was effectively refined through the addition of the Al-5Ti-0.25C master alloy, which enabled the formation of Al_3_Ti and TiC intermetallics. These intermetallics served as nucleation points during the solidification process of the Al matrix. Both refined and unrefined open-cell Al foams were produced with two distinct pore sizes.
- The refined samples exhibited a finer as-cast microstructure compared to their unrefined counterparts. The recrystallization behavior of the analyzed foams was assessed through annealing at different times.
- The optimal conditions were identified as 480 °C for 504 h, improving the microstructural homogeneity. The refined foams showed a 15% increase in mechanical strength and a 30% increase in energy absorption capacity, respectively, compared to unrefined foams.
- This enhancement was attributed to the grain refinement effects resulting from the presence of the TiC intermetallic and Al_3_Ti phases. Notably, the addition of Al-5Ti-0.25C to the foams, along with subsequent annealing, exhibited a 35% improvement in mechanical properties.
- The findings presented in this work support the development of mechanically enhanced open-cell aluminum foams suitable for applications in shock absorption and damping.

## Data Availability

The original contributions presented in this study are included in the article. Further inquiries can be directed to the corresponding authors.
